# Incarceration exposure during pregnancy and maternal disability: findings from the Pregnancy Risk Assessment Monitoring System

**DOI:** 10.1186/s12889-022-13143-7

**Published:** 2022-04-13

**Authors:** Alexander Testa, Chantal Fahmy, Dylan B. Jackson, Kyle T. Ganson, Jason M. Nagata

**Affiliations:** 1 Department of Management, Policy and Community Health, University of Texas Health Science Center at Houston, Houston, Texas US; 2 Department of Criminology & Criminal Justice, University of Texas at San Antonio, San Antonio, Texas US; 3 Johns Hopkins Bloomberg School of Public Health, Johns Hopkins University, Baltimore, US; 4 Factor-Inwentash Faculty of Social Work, University of Toronto, Toronto, Canada; 5 Department of Pediatrics, University of California, San Francisco, US

## Abstract

**Background:**

Extant research reveals that currently and formerly incarcerated individuals exhibit higher rates of disability. Moreover, recent research highlights that women exposed to incarceration during pregnancy —either personally or vicariously through a partner— face poorer health. However, prior research has not detailed the connection between incarceration exposure and risk for maternal disability.

**Methods:**

The aim of this study is to evaluate the association between a women’s exposure to incarceration during pregnancy and disability including difficulty with: communication, hearing, remembering, seeing, self-care, or walking. Data are from Pregnancy Risk Assessment Monitoring System (PRAMS), 2019 (*N* = 12,712). Logistic and negative binomial regression were used to assess the relationship between incarceration exposure and maternal disability.

**Results:**

Among the sample of women who delivered a recent live birth, approximately 3.3% of the sample indicated they were personally or vicariously exposed to incarceration in the 12 months before birth. Compared to those who did not have incarceration exposure, women with incarceration exposure have elevated odds of several disabilities, including difficulty remembering (Adjusted Odds Ratio [AOR] = 1.971; 95% Confidence Interval [CI] = 1.429, 2.718), difficulty seeing (AOR = 1.642, 95% CI = 1.179, 2.288), difficulty walking (AOR = 1.896, 95% CI = 1.413, 2.544), and a greater number of cumulative disabilities (Incidence Risk Ratio [IRR] = 1.483; 95% CI = 1.271, 1.731).

**Conclusions:**

Women personally or vicariously exposed to incarceration during pregnancy endure greater odds of having a disability. Considering both incarceration and disability are important public health issues with implications for maternal and child well-being, these findings highlight the need for further research that can better understand the connection between incarceration and disability.

**Supplementary Information:**

The online version contains supplementary material available at 10.1186/s12889-022-13143-7.

## Background

Approximately 61 million Americans are living with at least one disability. Among them, women report a higher prevalence of several functional disability types, including serious difficulty with vision, cognition, mobility, or communication [1]. Roughly 10–12% of women of reproductive age report having a disability, of which the majority have experienced a live birth [2–4]. Rates of adverse pregnancy and birth outcomes are elevated among women with disabilities across a wide range of measures [2, 5], including elevated rates of pre-eclampsia among women with developmental disabilities, and greater risk for low infant birth weight and early labor among women with physical disabilities[5].

Although research regarding both disabilities and reproductive health in the U.S. have grown in the past few decades, little is known about the distribution of disabilities among pregnant women and recent mothers across social strata [2]. Well-being during the perinatal period is closely intertwined with social determinants of health, and share many of the same disparities across the population [6]. Incarceration adds to these stark inequalities [7]. In the past five decades, the U.S. increased its incarceration rate faster than any other developed democracy in the world [8], resulting in significant consequences for population health [9, 10] and an ever-growing population of affected pregnant women and mothers [11]. Indeed, whether the woman is exposed to incarceration herself or vicariously through a partner, deleterious health effects abound, yielding a high risk of poorer maternal and infant health outcomes [12–17]. Moreover, a separate research literature finds that disabilities are more prevalent among adults who have been incarcerated or on community supervision [18–21], warranting recent calls to frame incarceration as a disability issue [22, 23]. However, despite research showing a connection between incarceration exposure and adverse maternal health, as well as a link between incarceration and disability more generally, no research to date has assessed the possible connection between incarceration exposure during pregnancy and maternal disability.

This is a notable gap considering incarceration-exposed pregnant women must reckon with considerable challenges related to their or their partner’s incarceration *and* they may have to do so while contending with multiple disabilities. Moreover, considering both incarceration [7, 8, 24] and disability [2, 25] are public health issues alongside the common reproductive issues facing many women in the U.S., greater insight of the interconnections between incarceration exposure and disability can enhance precision for targeted services to meet the needs of recent mothers and promote health equity. To address this gap, the current study examines the connection between incarceration and six forms of functional disabilities among a large sample of recently pregnant women.

### Methods

#### Data

Data are from the Pregnancy Risk Assessment Monitoring System (PRAMS), 2019. PRAMS is a population-based surveillance system conducted annually by the Centers for Disease Control and Prevention and state health departments. Using birth certificate records, participating states conduct a stratified sample of recent mothers who delivered a live birth in a given year. Women with multiple births are sampled at the same rate as those with singleton births [26]. Participating states collect data from a stratified systematic sample of 100 to 250 live births each month from a sampling frame of birth certificates. Data are collected from birth certificates data, state and territories’ vital record systems, and survey responses via a mailed questionnaire sent to recent mothers 2 to 4 months after delivery. Regarding the survey, a series of three mailing attempts are made 7–14 days after the previous attempt, and non-responders are followed up with up to 15 phone calls made over a 2-to-3-week period following the last mailing attempt. Sites are included in the PRAMS survey only if a minimum response threshold has been met. Since 2018, the minimum response threshold is 50 percent. The survey is accompanied with an introductory letter and an informed consent sheet.

Because birth certificate records are available for both responders and non-responders, information from non-responders is used to adjust for non-response rates. Moreover, birth certificate files are compared with the PRAMS sampling frame to adjust for non-coverage. Overall, the PRAMS annual sample is large enough to estimate site-wide risk factor proportions within 3.5% at 95% confidence. Additional information on the PRAMS survey and methodology can be found in reports by Shulman et al. [26] and Centers for Disease Control and Prevention (CDC) [27].

The PRAMS survey is based on a set of questionnaires that determined the analytic sample for the current study. A set of core questions are asked to all the sites, which include questions about the following topics: attitudes about the pregnancy, preconception care, prenatal care, Medicaid and WIC participation, breastfeeding, cigarette and alcohol use, health insurance coverage, physical abuse, infant health care, and contraception use. The remaining questions (including on incarceration) are chosen by a subset of sites from a pretested list of standard questions developed by the CDC or developed by sites on their own. Finally, in select years short question supplements are developed to append to the regular PRAMS survey for interested sites. In 2019, PRAMS administered a disability questionnaire supplement that consists of items from the Washington Group Short Set of Questions on Disability (WG-Short Set) [28] and are based upon the World Health Organization’s *International Classification of Functioning, Disability, and Health *(ICF) [29]. In total, 25 sites added the disability questionnaire supplement to their survey in 2019 [2]. The questions included on the PRAMS disability supplement can be found at: https://www.cdc.gov/prams/pdf/questionnaire/Disability-Supplement_508.pdf. The WG-SS has been validated in fifteen countries in Central America, South America, Asia, and Africa [30]. Additionally, while new to PRAMS, the WG-SS has been validated in perinatal and postnatal populations of women [31, 32].

**Fig. 1:**
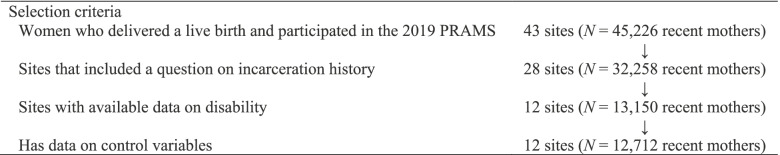
Sample selection flow chart for the Pregnancy Risk Assessment Monitoring System, 2019

The current study consists of 12 sites that included questions about both maternal disability and incarceration exposure, resulting in an analytic sample of 12,712 mothers (see Appendix A). Respondents in the study provided informed consent to participate in the PRAMS study. Sample selection based on availability of the measure of incarceration exposure, disability, and control variables is presented in Fig. 1. As detailed in Fig. 1, a total of 43 sites participated in the 2019 PRAMS. Among those, 28 sites asked about incarceration history, and 12 sites asked about both incarceration and disability. Among respondents in the 16 sites that asked about incarceration but not disability, compared to the 12 sites that asked about both incarceration and disability, there was no significant difference in the percentage of respondents who indicated they had incarceration exposure in the 12 months prior to birth (3.4% vs. 3.6%, *p* = 0.308). The use of the PRAMS data for this study was approved by the CDC PRAMS staff as part of the external researcher data sharing agreement: https://www.cdc.gov/prams/pdf/funding-opportunities/PRAMS-Data-Sharing-Agreement_508-tagged.pdf.

### Independent variable

#### Incarceration exposure

Incarceration exposure is a binary variable measured using a survey item that asks a respondent whether in the 12 months prior to birth, “I or my husband or partner went to jail” (0 = no; 1 = yes) [33, 34]. The use of a single item self-reported question about incarceration history is common in incarceration research using large-scale survey data [35, 36]. The question has also been used as the basis of research using PRAMS data for over a decade [13, 16, 17, 33, 37, 38]. It is important to note that because the question conflates the incarceration of a mother or her husband/partner, we cannot discern who experienced the incarceration. Even so, there is good reason to consider that in most cases it refers to the incarceration of a male partner. First, approximately 93 percent of the prison population [39], and about 85 percent of the jail population in the United States are males [40]. Second, relatively few women are pregnant upon admission to prison  [41]. Third, because the PRAMS data ask about incarceration in the 12 months prior to birth, and the survey is completed by those in the general population (i.e., not incarcerated) approximately 2–4 months following birth, for a woman to be incarcerated during pregnancy, there is a narrow window during which the incarceration could have occurred. Finally, it is important to note that the question inquires about incarceration in jail. However, consistent with prior research [17, 33, 34], this measure is used as a proxy for incarceration in any type of correctional facility, considering that the terms jail and prison are often used interchangeably among the general public.

### Dependent variables

The focal outcome variables are self-reports of six maternal functional disabilities from the WG-Short Set, which inquire about current disabilities at the time the survey is taken (approximately 2–4 months following birth): [2] (1) *communication* (“Using your usual language, do you have difficulty communicating, for example, understanding, or being understood?”), (2) *hearing* (“Do you have difficulty hearing, even if using a hearing aid(s)?”), (3) *cognition* (“Do you have difficulty remembering or concentrating?”), (4) *vision* (“Do you have difficulty seeing, even when wearing glasses or contact lenses?”), (5) *mobility* (“Do you have difficulty walking or climbing steps?”, and (6) *self-care *(“Do you have difficulty with self-care, such as washing all over or dressing?”). Responses included (a) no difficulty, (b) some difficulty, (c) a lot of difficulty, and (d) I cannot do this at all. Responses were recoded into dichotomous variables (0 = no difficulty, 1 = any difficulty with the activity). In addition, we calculated the cumulative number of disabilities by summing the six types of disabilities into a single index. The decision to dichotomize the outcome variables was driven by two factors. First, across each of the six disability measures, few respondents (often < 1% of the sample) replied that they had a lot of difficulty or that they could not do the activity at all. The percentage of respondents indicating their disability status based on the original response categories prior to dichotomization is detailed in Appendix B. Second, dichotomization is consistent with recent PRAMS research using the disability supplement [42].

### Control variables

Consistent with prior research using PRAMS data, the current study includes several control variables that account for demographic, socioeconomic, and health related characteristics that may be related to both incarceration and disability status [42, 43]. The control variables include *maternal age* (17 or younger, 18–24, 25–29, 30–34, and 35 or older), *maternal race* (White, Hispanic, Black, Native American, Asian or Other), *maternal educational attainment* (0 = less than college; 1 = college graduate), *marital status* (0 = not currently married; 1 = currently married), *body mass index classification* (underweight, normal weight, overweight, obese), *household income* (≤ $16,000, $16,0001-$40,000, $40,001-$85,000, > $85,000), *number of financial dependents* (range 0–7), and *state of residence*.

### Statistical analysis

The association between incarceration exposure and the six individual forms of disabilities are assessed using logistic regression. Negative binomial regression is used to examine the relationship between incarceration exposure and the cumulative number of disabilities considering the large number of zero values and strong positive skew (i.e., overdispersed). Analyses are conducted using Stata version 16.1. All models were adjusted for survey weights and strata information to account for the complex survey design of PRAMS. Patterns of missing data on variables in the 12 sites included in the analysis is reported in Appendix C.

## Results

Table 1 presents the summary statistics of the analytic sample stratified by incarceration exposure. About 3.4% (*n* = 426) of the sample reported exposure to incarceration in the 12 months prior to birth. Compared to recent mothers without incarceration exposure, mothers with incarceration exposure reported significantly higher rates of difficulty communicating (10.8% vs. 4.2%, *p* = 0.005), difficulty remembering (50.0% vs. 27.1%, *p* < 0.001), difficulty seeing (34.9% vs. 18.6%, *p* < 0.001), difficulty walking (16.2% vs. 5.1%, *p* < 0.001), as well as more total disabilities on average (1.22 vs. 0.62, *p* < 0.001).

**Table 1:** Summary statistics of analytic sample stratified by incarceration exposure—pregnancy risk assessment monitoring system (PRAMS), 2019 (*N* = 12,712)

**Variables**	**No Incarceration**	**Incarceration**	
	**(*N* = 12,276)**	**(*N* = 426)**	
	**% / Mean (SD)**	**% / Mean (SD)**	***p-value***
*Disability Type*			
Communication	4.2%	10.8%	.005
Hearing	4.6%	6.7%	.163
Cognition	27.1%	50.0%	< .001
Vision	18.6%	34.9%	< .001
Self-care	2.0%	3.1%	.162
Mobility	5.1%	16.9%	< .001
Total disabilities	.62 (0.01)	1.22 (0.09)	< .001
*Maternal Age*			
< 18	0.6%	1.7%	.109
18–24	20.2%	35.8%	< .001
25–29	29.7%	39.5%	.019
30–34	31.2%	12.3%	< .001
35 +	18.3%	10.7%	.002
*Maternal Race/Ethnicity*			
White	62.9%	52.8%	.013
Hispanic	13.0%	12.2%	.817
Black	17.4%	29.0%	< .001
Other Race/Ethnicity	6.7%	6.0%	.704
Currently Married	62.7%	18.4%	< .001
*Maternal Educational Attainment*			
Less than High School	9.6%	27.2%	< .001
High School Graduate	24.1%	32.7%	.014
Some College	27.0%	35.5%	.023
College Graduate	39.3%	4.5%	< .001
*Body Mass Index*			
Underweight	2.9%	7.6%	.162
Normal Weight	40.9%	39.9%	.803
Overweight	26.1%	26.0%	.968
Obese	30.1%	26.5%	.304
*Household Income*			
< $16,000	18.4%	67.2%	< .001
$16,000-$40,000	21.4%	19.4%	.476
$40,001-$85,000	30.3%	12.2%	< .001
> 85,000	29.9%	1.2%	< .001
Number of Dependents	2.94 (0.02)	2.97 (0.12)	.735

Table 2 presents the results of the regression analysis of disabilities on incarceration exposure, adjusting for the inclusion of control variables. Findings show that incarceration-exposed women were significantly more likely to report several disabilities including difficulty remembering (Adjusted Odds Ratio [AOR] = 1.971; 95% Confidence Interval [CI] = 1.429, 2.718), difficulty seeing (AOR = 1.642, 95% CI = 1.179, 2.288), and difficulty walking (AOR = 1.896, 95% CI = 1.413, 2.544). Expressed as predicted probabilities, these results translate to incarceration-exposed women being about 15 percentage points more likely to have difficulty remembering (41.3% vs. 26.3%), 8.3 percentage points more likely to have difficulty seeing (25.9% vs. 17.6%), and nearly 4 percentage points more likely to report difficulty walking (8.5% vs. 4.7%). Finally, the results in model 7 show that incarceration-exposed women incurred a significantly higher rate of total disabilities relative to women without incarceration exposure (Incidence Risk Ratio [IRR] = 1.483; 95% CI = 1.271, 1.731).

**Table 2:** Results of logistic regression and negative binomial regression of individual and cumulative disabilities on incarceration exposure—pregnancy risk assessment monitoring system (PRAMS), 2019 (*N* = 12,712)

	**Model 1: Difficulty Communicating**	**Model 2: Difficulty Hearing**	**Model 3: Difficulty Remembering**	**Model 4: Difficulty Seeing**	**Model 5: Difficulty Self Care**	**Model 6: Difficulty Walking**	**Model 7: Cumulative Disabilities**
	**AOR 95% CI**	**AOR 95% CI**	**AOR 95% CI**	**AOR 95% CI**	**AOR 95% CI**	**AOR 95% CI**	**IRR 95% CI**
Incarceration Exposure	1.492	1.275	1.971***	1.642**	0.869	1.896***	1.483***
	(0.869—2.564)	(0.775—2.096)	(1.429—2.718)	(1.179—2.288)	(0.460—1.639)	(1.413—2.544)	(1.271—1.731)

Control variables include: maternal age, maternal race/ethnicity, maternal educational attainment, marital status, body mass index, household income, number of financial dependents, and state of residence

*Abbreviations: AOR* adjusted odds ratio, *IRR* incidence rate ratio

^***^p < 0.001, **p < 0.01

A sensitivity analysis for unmeasured confounding in observational studies was performed using the methodology proposed by VanderWeele and Ding [44]. Analyses were conducted using the “evalue” package in Stata [45]. The results for the point estimate and confidence interval are presented in Appendix D. As an interpretation, the point estimate for the “difficulty remembering” outcome suggests that the observed odds ratio could be explained away by an unmeasured confounder that was associated with both incarceration and difficulty remembering by a risk ratio of 3.354 and beyond. The confidence interval row suggests that an unmeasured confounder associated with difficulty remembering and incarceration by a risk ratio of 2.212 or beyond could explain away the lower confidence limit. Overall, the results of the analysis suggest that the findings are moderately robust [45]. Finally, the results were also assessed using multiple imputation with chained equations (20 multiply imputed datasets). The findings of this analysis provided substantively similar findings as the main analysis using listwise deletion, indicating that the findings remain robust with different procedures of handling missing data (results available upon request).

## Discussion

The findings of the current study revealed that incarceration-exposed women were significantly more likely to experience a variety of disabilities including difficulty remembering, difficulty seeing, difficulty walking, as well as reported a greater rate of disabilities overall. These results add to previous literature that has demonstrated higher levels of disability among incarceration-exposed populations [19–21], as well as research documenting the consequences of incarceration exposure for women’s health [11, 17].

Although the current study is not able to establish the exact mechanisms of why recent mothers who were exposed to incarceration in the 12 months prior to birth incur higher incidence of certain forms of disability, there are a few potential possibilities worth discussing. First, personal—rather than vicarious—incarceration may be related to the prevalence of disabilities due to a physical injury lacking medical attention, as data from U.S. jails show that nearly one in eight persons reported an injury from an accident or fighting since admission [46]. An incarcerated woman may incur an injury that is neglected during the course of imprisonment and upon release may continue to ignore clear signs that medical attention is required. When disregarded, it may worsen, causing irreparable damage in the form of a functional disability. Second, if a woman’s partner is exposed to incarceration, the psychological strain and distress of having a loved one incarcerated may engender adverse coping mechanisms [47], by which psychiatric disorders may arise. Whether in the case of personal or vicarious incarceration, the overlap between psychiatric-related disorders and disability is compelling. It does not matter if the mental illness began before, during, or after the incarceration term [21, 48]. In fact, Schnittker and colleagues [21] find psychiatric disorders largely mediates the association between incarceration and both mobility and cognitive disability, leading these scholars to suggest “that disability differences between former inmates and others could be reduced greatly by addressing psychiatric disorders.” Thus, it is possible that psychiatric disorders may underlie the association between incarceration and several functional disabilities, found in this study—especially disabilities related to communication, cognition, and self-care. Relatedly, stress can manifest in physical health conditions, including chronic health problems [49–51]. To be sure, research has showed significant declines in women’s cardiovascular health due to the stress of having a family member incarcerated [52, 53]. In addition, recent scholarship has shown that due in part to stress, mothers endure greater levels of functional impairments after the incarceration of a child [54, 55]. Accordingly, it is possible that the advent of partner’s incarceration during pregnancy may elevate the presence of functional disabilities in a woman. Lastly, gender, race, and disability play a significant role in incarceration rates as a sizable proportion of prisoners are poor people of color [56]. Living in poverty causes a variety of impairments linked to disabling conditions. And, once incarcerated, the prison environment itself is disabling [56]. Thus, guided by this framework, disability relates more to the social and economic conditions that lead people to both a higher chance of disablement and imprisonment, evincing a selection effect [56]. As Wildeman and Lee’s [11] recent review of research on incarceration and women’s health concluded: “we can say that women who have had a family member incarcerated tend to be sicker than women who haven't, but we cannot tell for sure whether family member incarceration partially causes poor health.”

The findings hold several implications for improving the health and well-being for incarceration-exposed women as well as extending resources to mothers with disabilities. For instance, health care providers should consider both personal and vicarious contact with the carceral system as a risk factor for disability. In cases where a woman is incarcerated herself during pregnancy, it would be beneficial for medical staff within the correctional facility to screen for disabilities and provide appropriate support, as well as connect women to community resources and medical care following release. In situations where a woman is exposed to incarceration vicariously through a partner, it is likely that such a situation will result in immense social, familial, and health challenges during pregnancy and following birth [57]. In cases when the mother is also experiencing disability and an incarcerated partner, the challenges they face are likely augmented. A beneficial avenue can be to ask their male partners during prison intake or upon release if they have a romantic partner who is pregnant and experiences disabilities to provide outreach and connect disabled women to appropriate services. Likewise, prison visitation waiting rooms can be unique opportunities for outreach to disabled pregnant women [57].

More broadly, policy initiatives can be implemented to better support the health needs of incarceration-exposed mothers. For instance, the Maternal and Child Health Bureau (MCHB) supports children and families with disabilities, yet does not have active programs supporting prospective or recent mothers with disabilities [25]. A fruitful avenue is for programs such as MCHB to work with state and local agencies and community organizations to provide resources to disabled women and mothers. Finally, targeted home visiting programs such as Nurse Family Partnership, which provides nurses to meet with new mothers on a regular basis both during pregnancy and in the years following birth, could be a potentially useful intervention for incarceration-exposed women who are facing the dual challenges of the removal of a partner via incarceration and a current disability [58].

## Limitations

The current study has several limitations that can be expanded upon by future research. First, the question measuring incarceration exposure in the PRAMS survey does not differentiate between a woman’s partner’s incarceration or her own. Based on national data, we surmise that in most cases this corresponds with a male partner, as only 15% of the jail population and only 7% of the prison population are female [40, 59]. It is important for future work to assess how disability status may differ in the case when a woman experiences incarceration compared to if this experience is vicarious through a partner. Second, the measure of incarceration is related to the 12 months before birth, and therefore we cannot capture lifetime exposure to incarceration. Third, the incarceration exposure is a dichotomous variable and lacks information related to potentially important features of the incarceration experience such as the duration of incarceration or the length of time since release. Fourth, based on the wording of the survey question in the PRAMS data, we cannot determine whether an individual was incarcerated in prison or jail. Accordingly, it is possible that some individuals who were incarcerated in prison may have answered “no” to the incarceration question. Even so, if this is the case, this would downwardly bias the findings and suggest the estimates in the current study are conservative. Fifth, disabilities are self-reported and therefore do not represent a medical diagnosis and have not been confirmed or validated against other data. Future research that uses diagnoses by a health professional may be valuable. Sixth, the PRAMS data are cross-sectional data from a single year and therefore, cannot establish causality. Finally, there are potentially relevant confounding variables that were not available in the PRAMS data including maternal history of somatic or psychiatric disorders.

## Conclusion

Incarceration and disability are both significant public health issues. When mothers have experienced both life events, it can have substantial negative implications for both maternal and child well-being. Accordingly, fostering a greater understanding of the repercussions of both incarceration and disability, as well as the interconnection between the two for pregnant women and mothers, is essential for supporting the design and implementation of programs and interventions that promote greater health equality among this vulnerable population.

## Supplementary Information

**Additional file1:** **Appendix A. **States in analytic sample. **Appendix B. **Percentage of responses to severity of disability. **Appendix C. **Percentage of missing data on variables in analysis. **Appendix D. **Sensitivity analysis of unmeasured confounding.

## Data Availability

The datasets generated and/or analyzed during the current study are not publicly available due to the nature of Pregnancy Risk Assessment Monitoring System not being publicly available. Data used in this study can be requested at https://www.cdc.gov/prams/index.htm. Queries about the data can be directed to Alexander Testa: alexander.testa@utsa.edu.
