# Supplementary material for: Incarceration exposure during pregnancy and maternal disability: findings from the Pregnancy Risk Assessment Monitoring System
Source: BMC Public Health. 2022 Apr 13;22:744. doi: 10.1186/s12889-022-13143-7 (PMC9009053; doi:10.1186/s12889-022-13143-7)
Supplement: Supplementary file 1 — Additional file1: Appendix A. States in analytic sample. Appendix B. Percentage of responses to severity of disability. Appendix C. Percentage of missing data on variables in analysis. Appendix D. Sensitivity analysis of unmeasured confounding. [file 12889_2022_13143_MOESM1_ESM.docx]

**Appendix A: States in Analytic Sample**

| **State** | **Frequency** | **Percentage** |
| --- | --- | --- |
| Colorado | 1,034 | 8.13 |
| Georgia | 655 | 5.15 |
| Kansas | 951 | 7.48 |
| Louisiana | 891 | 7.01 |
| Massachusetts | 1,528 | 12.02 |
| Maine | 576 | 4.53 |
| Michigan | 1,225 | 9.64 |
| Missouri | 1,477 | 11.62 |
| Mississippi | 1,108 | 8.72 |
| Nebraska | 1,462 | 11.5 |
| New York | 701 | 5.51 |
| Oregon | 1,104 | 8.68 |
| **Total** | **12,712** | **100** |

**Appendix B: Percentage of Responses to Severity of Disability**

|  | **Difficulty Communicating** | **Difficulty Hearing** | **Difficulty Remembering** | **Difficulty Seeing** | **Difficulty Self Care** | **Difficulty Walking** |
| --- | --- | --- | --- | --- | --- | --- |
| No difficulty | 94.82% | 95.28% | 71.56% | 80.47% | 97.61% | 93.90% |
| Some difficulty | 4.46% | 4.12% | 24.51% | 17.63% | 2.06% | 5.24% |
| A lot of difficulty | 0.56% | 0.25% | 3.79%. | 1.59% | 0.20% | 0.71% |
| I cannot do this at all | 0.16% | 0.35% | 0.14% | 0.31% | 0.12% | 0.15% |

**Appendix C: Percentage of Missing Data on Variables in Analysis**

| **Variables** | **Percent Missing** |
| --- | --- |
| Disability Type | 3.3% |
| Incarceration Exposure | 1.6% |
| Maternal Age | 0% |
| Maternal Race/Ethnicity | 0.28% |
| Currently Married | 0.08% |
| Maternal Educational Attainment | 0.65% |
| Body Mass Index | 0% |
| Household Income | 7.32% |
| Number of Dependents | 3.8% |

**Appendix D: Sensitivity Analysis of Unmeasured Confounding**

|  | **Difficulty Remembering** | **Difficulty Seeing** | **Difficulty Walking** | **Cumulative Disabilities** |
| --- | --- | --- | --- | --- |
| Point Estimate | 3.354 | 2.669 | 3.119 | 2.327 |
| Confidence Interval | 2.212 | 1.638 | 2.177 | 1.858 |
